# Surgical management of endometrial cancer: evidence-based principles and recommendations: Number 15 – 2026

**DOI:** 10.61622/rbgo/2026FPS15

**Published:** 2026-10-13

**Authors:** Agnaldo Lopes da Silva, Eduardo Batista Cândido, Jesus Paula Carvalho, Caetano da Silva Cardial, Matheus Eduardo Soares Pinhati, Mariana Seabra Leite Praça

**Affiliations:** Universidade Federal de Minas Gerais Belo Horizonte MG Brazil Universidade Federal de Minas Gerais, Belo Horizonte, MG, Brazil; Universidade Federal de Minas Gerais Belo Horizonte MG Brazil Universidade Federal de Minas Gerais, Belo Horizonte, MG, Brazil; Faculdade de Ciências Médicas Belo Horizonte MG Brazil Faculdade de Ciências Médicas, Belo Horizonte, MG, Brazil.; Universidade de São Paulo Faculty of Medicine São Paulo SP Brazil Faculty of Medicine, Universidade de São Paulo, São Paulo, SP, Brazil.; Centro Universitário FMABC Santo André SP Brazil Centro Universitário FMABC, Santo André, SP, Brazil.; Universidade Estadual Paulista Botucatu SP Brazil Universidade Estadual Paulista, Botucatu, SP, Brazil.; Universidade Federal de Minas Gerais Belo Horizonte MG Brazil Universidade Federal de Minas Gerais, Belo Horizonte, MG, Brazil

## Key Points

- Endometrial cancer (EC) is the most prevalent gynecologic malignancy in high-income nations, with incidence rates rising due to aging and obesity.
- Surgery is the primary therapeutic modality, providing essential data for staging, risk stratification, and adjuvant planning.
- Minimally invasive surgery (MIS) provides equivalent oncologic outcomes to open surgery while significantly reducing perioperative morbidity and recovery time.
- Sentinel lymph node (SLN) mapping offers high diagnostic accuracy and has replaced systematic lymphadenectomy as the preferred method for nodal assessment.

## Recommendations

- Patients with suspected or confirmed endometrial cancer should undergo systematic initial assessment including: comprehensive clinical assessment, histopathologic confirmation, and appropriate imaging based on clinical and pathologic risk.
- Preoperative risk stratification should integrate clinical characteristics, imaging findings, histopathologic features, and molecular classification whenever available.
- Minimally invasive total hysterectomy with bilateral salpingo-oophorectomy (TH/BSO) should be offered as the standard surgical approach for eligible patients with apparent early-stage disease.
- Sentinel lymph node mapping should be the preferred method for lymph node assessment. Systematic lymphadenectomy should be performed in suspected lymph node metastases, but not in uterine-confined disease.
- Molecular tumor classification, including assessment of mismatch repair genes status, should be performed in all patients to inform prognosis, hereditary risk, and adjuvant treatment decisions.
- Fertility-sparing treatment should be restricted to highly selected patients with strictly low-risk disease and managed in specialized centers with strict surveillance.
- Patients who are not candidates for surgery should be managed with radiotherapy, systemic therapy, hormonal treatment, or combined approaches according to disease extent and tumor biology.
- Surgical treatment of advanced or recurrent endometrial cancer should be reserved for carefully selected patients in whom complete cytoreduction or meaningful palliation is achievable.
- Multidisciplinary decision-making is essential at all stages of endometrial cancer management to optimize oncologic outcomes and preserve quality of life.

## Background

Endometrial cancer is the most common gynecologic malignancy in high-income countries, with a steadily increasing global incidence driven by population aging and rising obesity rates. It accounts for approximately 4.5% of all cancers in women, with more than 400,000 new cases and nearly 100,000 deaths reported annually worldwide. Although most patients are diagnosed at an early stage, disparities in access to care and a higher prevalence of aggressive histologic and molecular subtypes contribute to worse outcomes, particularly in low- and middle-income regions.^(1,2)^

Surgery remains the cornerstone of endometrial cancer management and plays a central role in diagnosis, staging, prognostic stratification, and treatment planning. TH/BSO represents the standard initial treatment for most patients, and surgical staging is essential to guide adjuvant therapy decisions, as emphasized by major international guidelines.^(1,3–5)^ In carefully selected premenopausal women with low-risk, early-stage disease, ovarian preservation may be considered to avoid surgical menopause without compromising oncologic outcomes.^(1,4)^

Over the past two decades, the surgical approach to endometrial cancer has evolved substantially. Minimally invasive surgery, including laparoscopic and robotic-assisted techniques, has become the preferred approach for apparent early-stage disease, supported by robust evidence demonstrating equivalent oncologic outcomes compared with open surgery, along with reduced perioperative morbidity, shorter hospital stay, and faster recovery.^(1,6,7)^ These advantages are particularly relevant given the high prevalence of obesity and comorbidities among patients with endometrial cancer.^(6–8)^

Similarly, lymph node assessment has shifted from systematic pelvic and para-aortic lymphadenectomy toward sentinel lymph node mapping. Randomized trials and meta-analyses have shown no survival benefit from systematic lymphadenectomy in uterine-confined disease, while sentinel lymph node biopsy provides accurate staging with significantly lower morbidity.^(1,9)^ As a result, sentinel lymph node mapping is now recommended by international guidelines for most patients undergoing surgical staging.^(1,3,9)^

More recently, the incorporation of histopathologic and molecular factors into staging and risk stratification has further refined surgical decision-making. The updated FIGO staging system and ESGO-ESTRO-ESP guidelines integrate histologic subtype, lymphovascular space invasion, and molecular classification to support individualized, risk-adapted management strategies.^(3,10)^

In this context, the objective of this Position Statement is to synthesize the current evidence and provide practical, consensus-based guidance on the surgical management of endometrial cancer, emphasizing evidence-based principles, appropriate patient selection, and multidisciplinary care to optimize oncologic outcomes while minimizing treatment-related morbidity. To facilitate clinical application, an integrated surgical decision-making roadmap has been developed in figure 1.

**Figure 1:**
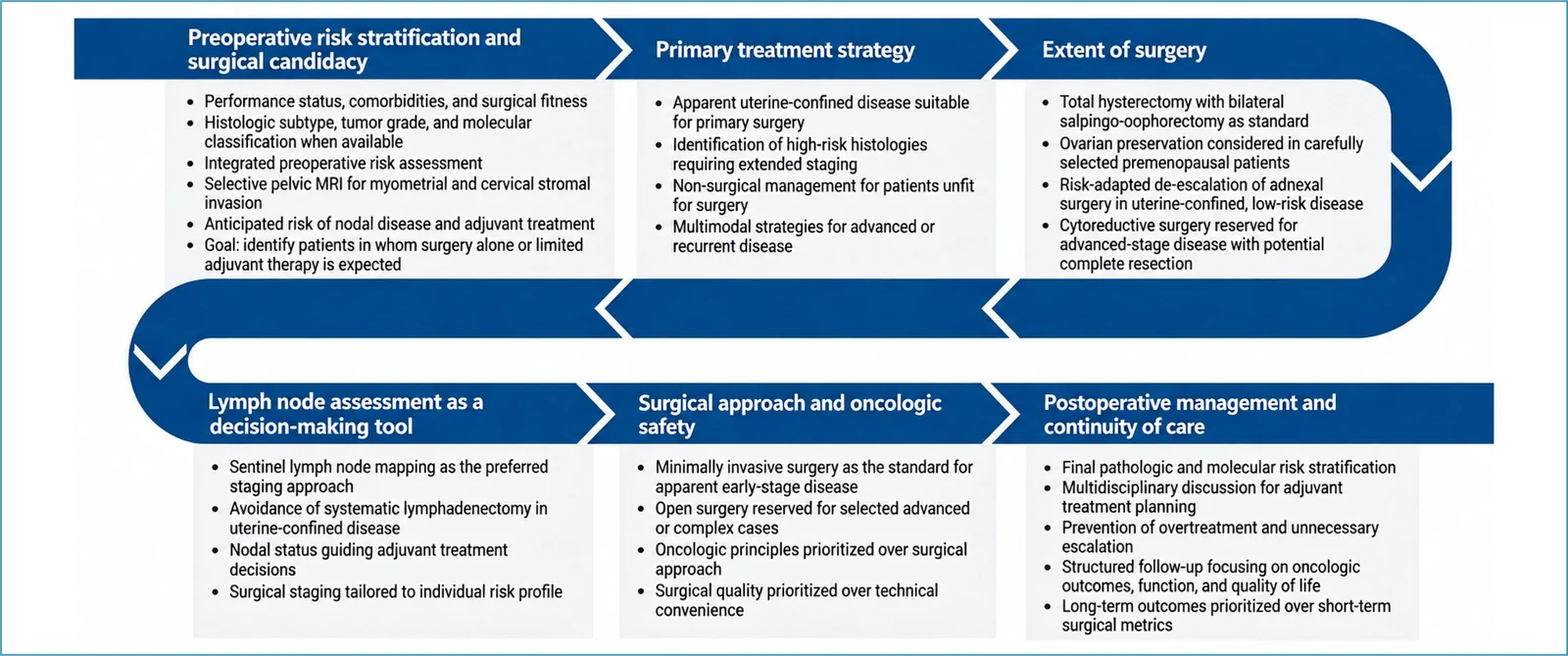
Surgical decision-making roadmap for patients with endometrial cancer eligible for surgical treatment

This tool delineates the continuum of care, from initial risk stratification to postoperative surveillance, aligning with contemporary evidence-based principles. The core principles that underpin contemporary surgical management of endometrial cancer are summarized in figure 2.

**Figure 2:**
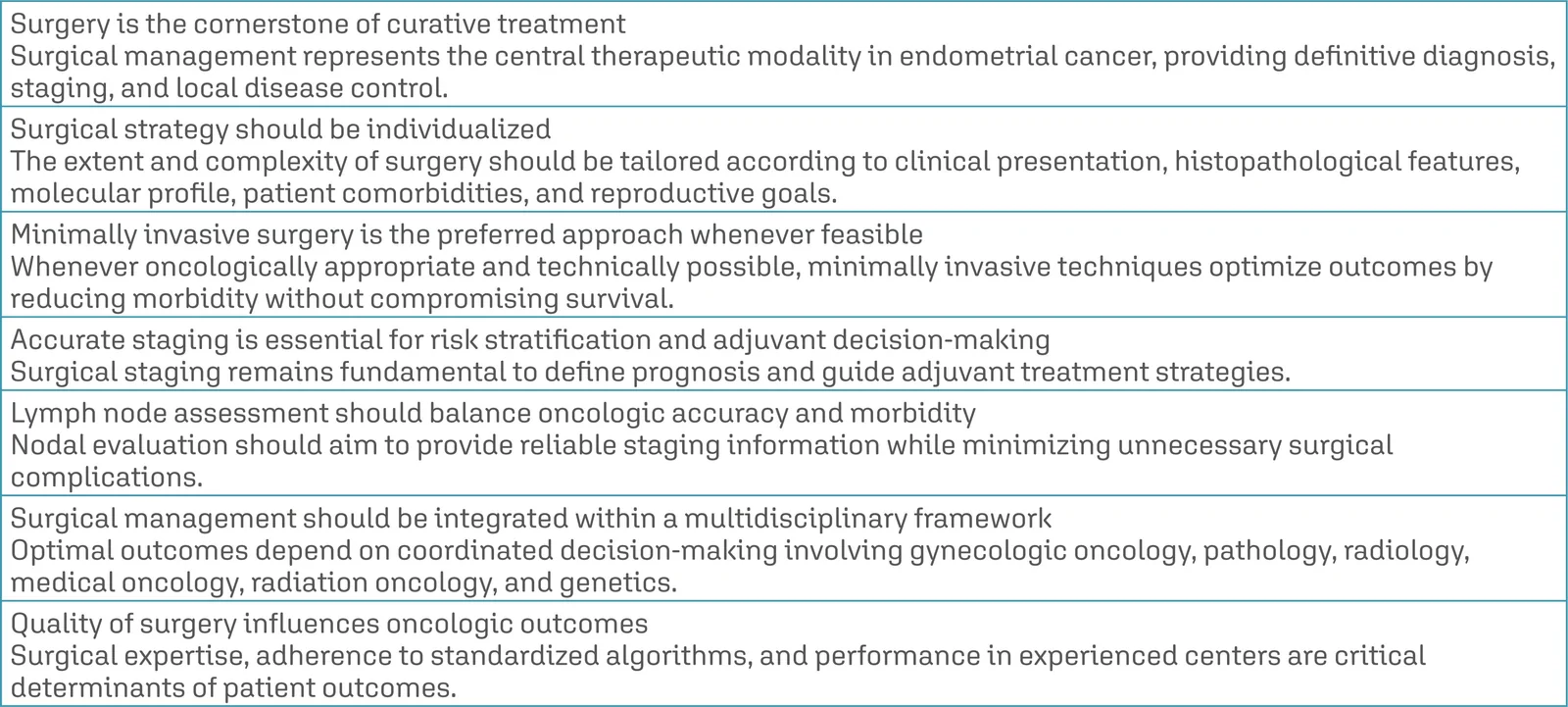
Core principles of surgical management in endometrial cancer

## What is the recommended initial assessment of patients with suspected or confirmed endometrial cancer?

The initial assessment of patients with suspected or confirmed endometrial cancer should be systematic and focused on confirming the diagnosis, evaluating disease extent, and identifying factors that may influence surgical planning. A detailed clinical history and physical examination are essential, with particular attention to abnormal uterine bleeding, menopausal status, age, obesity, metabolic comorbidities, and personal or family history suggestive of hereditary cancer syndromes.^(2,11)^

Transvaginal ultrasonography is the first-line imaging modality in the evaluation of abnormal uterine bleeding. In postmenopausal women, an endometrial thickness below 4 mm reliably excludes clinically significant disease, whereas increased thickness or persistent symptoms warrant further investigation. In premenopausal women, endometrial thickness has limited predictive value, and diagnostic evaluation should be guided primarily by clinical presentation.^(2,5,11)^

Histologic confirmation is mandatory for diagnosis. Endometrial sampling, most commonly performed using an office-based biopsy device, is the preferred initial diagnostic method. When sampling is inadequate or inconclusive, or when focal intrauterine lesions are suspected, hysteroscopy with directed biopsy or dilation and curettage is recommended.^(2,11)^

Once endometrial cancer is diagnosed, imaging plays a complementary role in preoperative assessment. Pelvic magnetic resonance imaging is recommended when evaluation of myometrial invasion, cervical stromal involvement, or fertility-sparing strategies may influence surgical planning. Cross-sectional imaging of the chest, abdomen, and pelvis (using computed tomography and, in selected cases, FDG-PET/CT) is indicated in high-grade tumors or when extrauterine disease is suspected. Routine advanced imaging is not required in clearly low-risk, uterine-confined disease.^(12)^

The results in the treatment of patients with endometrial carcinoma are better when carried out by specialists in reference centers, as specialist management is associated with improved staging accuracy, appropriate use of minimally invasive techniques, and optimized oncologic outcomes.^(2,3)^ The goal of the initial assessment is to establish a comprehensive clinical and pathologic framework that supports individualized, evidence-based surgical decision-making. The essential components of the preoperative assessment of patients with endometrial cancer who are candidates for surgery are summarized in figure 3.

**Figure 3:**
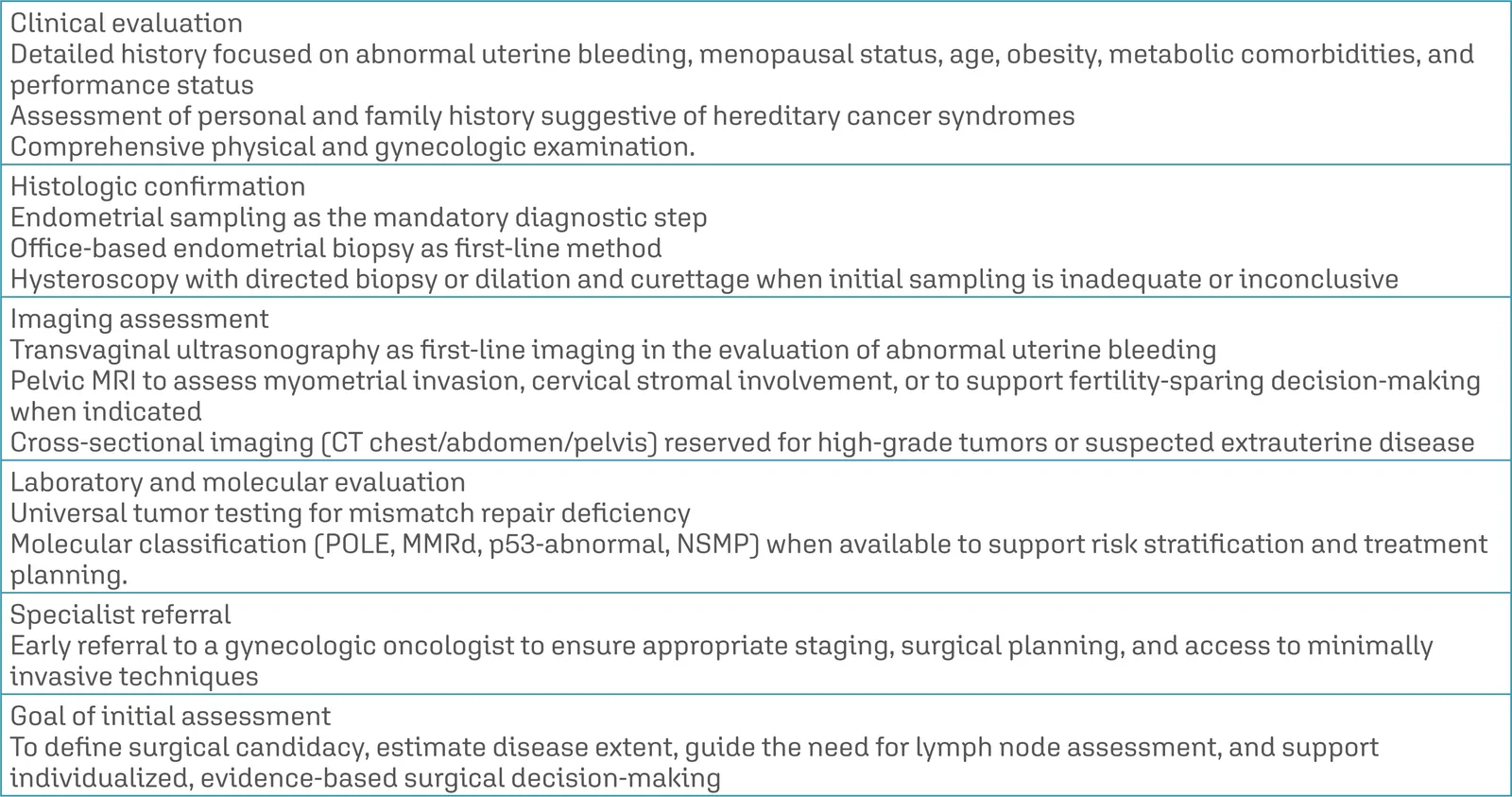
Essential preoperative assessment of patients with endometrial cancer eligible for surgery

## How should preoperative risk stratification be performed in endometrial cancer?

Preoperative risk stratification in endometrial cancer is essential to guide the extent of surgical staging, particularly lymph node assessment, and to inform multidisciplinary treatment planning. Risk stratification should be based on an integrated evaluation of patient-related factors, tumor characteristics, imaging findings, and molecular classification, rather than on isolated parameters.

Clinical characteristics associated with increased risk of advanced disease include older age, postmenopausal status, obesity (body mass index ≥35 kg/m²), and the presence of metabolic comorbidities such as diabetes mellitus and hypertension. These factors are consistently associated with a higher likelihood of occult carcinoma, deep myometrial invasion, and nodal involvement, and should be considered during surgical planning.^(13)^ Increased endometrial thickness on transvaginal ultrasonography, particularly values ≥14 mm, has been associated with higher tumor grade and stage and may further refine preoperative risk assessment, especially in patients initially diagnosed with premalignant lesions.^(13–15)^

Tumor-related features play a central role in risk stratification. Histopathologic parameters obtained from preoperative endometrial sampling, including tumor grade and histologic type, provide critical information. High-grade tumors and non-endometrioid histologies are associated with an increased risk of lymph node metastasis and extrauterine disease and generally warrant comprehensive surgical staging or sentinel lymph node assessment. Imaging findings, particularly the presence of deep myometrial invasion, cervical stromal involvement, or radiological suspicion of extrauterine spread, further stratify risk and influence the choice and extent of surgical intervention.^(1,5,14,16)^

Molecular classification has emerged as a key component of contemporary risk stratification in endometrial cancer. Incorporation of molecular subgroups (POLE-mutated, mismatch repair–deficient, p53-abnormal, and no specific molecular profile (NSMP)) significantly improves prognostic accuracy beyond traditional clinicopathologic models. Tumors with p53 abnormalities and mismatch repair deficiency are associated with a higher risk of extrauterine dissemination and recurrence, whereas POLE-mutated tumors demonstrate an excellent prognosis despite high-grade histologic features.^(16–20)^ Increasing evidence supports the integration of molecular data with clinical, histopathologic, and imaging findings to optimize preoperative risk assessment and enable more individualized surgical strategies.^(16,19,21,22)^

Overall, preoperative risk stratification in endometrial cancer should follow a multidimensional approach that combines patient characteristics, tumor pathology, imaging findings, and molecular classification.^(5)^ This integrated strategy allows for tailored surgical management, avoids overtreatment in low-risk patients, and supports appropriate staging and treatment intensification in those at higher risk.

## What is the role of molecular classification in endometrial cancer management?

Molecular classification has fundamentally transformed the management of endometrial cancer by shifting prognostic assessment and therapeutic decision-making from purely histopathologic criteria to biologically defined tumor subgroups. Current international consensus, endorsed by the World Health Organization and incorporated into major clinical guidelines, recognizes four principal molecular subtypes: POLE-mutated, mismatch repair–deficient (MMRd), p53-abnormal (p53abn), and no specific molecular profile (NSMP). These subtypes are identified using a combination of POLE gene sequencing and immunohistochemical assessment of mismatch repair proteins and p53, with estrogen receptor status providing additional prognostic refinement within the NSMP subgroup.^(1,5,17,23–25)^

Molecular subtyping provides superior prognostic discrimination compared with clinicopathologic models alone. Each molecular subgroup exhibits distinct biological behavior and clinical outcomes that are largely independent of histotype and grade. POLE-mutated tumors are associated with an excellent prognosis and low risk of recurrence, even in the presence of high-grade features, whereas p53-abnormal tumors demonstrate aggressive behavior, increased risk of extrauterine spread, and poorer survival. Tumors classified as MMRd and NSMP show intermediate and heterogeneous outcomes, with estrogen receptor status further refining risk stratification within the NSMP category.^(1,17–19,26)^

From a preoperative perspective, increasing evidence indicates that integrating molecular classification with clinical, imaging, and histopathologic parameters improves the accuracy of risk stratification compared with traditional models alone. Studies evaluating preoperative algorithms that incorporate molecular data demonstrate superior prediction of advanced stage disease and nodal involvement, particularly for identifying high-risk patients with p53-abnormal tumors.^(16,21)^ In this context, molecular classification outperforms histologic subtype and tumor grade as predictors of occult extrauterine disease when combined with imaging findings.^(16,21)^

Nevertheless, the impact of molecular classification on surgical decision-making remains evolving. Current international guidelines do not recommend the routine use of molecular classification alone to determine the extent of surgical staging, including lymphadenectomy or omentectomy. Surgical planning should continue to rely on an integrated assessment combining molecular subtype, imaging findings, and clinicopathologic features. The 2025 ESGO-ESTRO-ESP guidelines and the revised FIGO 2023 staging system acknowledge the prognostic relevance of molecular classification and support its incorporation into preoperative risk stratification when available, while emphasizing that prospective validation is still required before molecular features independently guide the extent of surgical staging.^(3,27–29)^

In contrast, the role of molecular classification in guiding adjuvant treatment is well established. Robust evidence supports treatment de-escalation for patients with POLE-mutated tumors due to their excellent prognosis, regardless of conventional risk factors. Conversely, patients with p53-abnormal tumors derive significant benefit from intensified adjuvant therapy, including combined chemoradiotherapy, as demonstrated in randomized clinical trials. MMRd tumors represent a biologically distinct subgroup with demonstrated sensitivity to immune checkpoint inhibition in advanced or recurrent disease. Management of NSMP tumors requires a more individualized approach, with estrogen receptor status influencing decisions regarding hormonal therapy versus chemoradiotherapy.^(25,30–32)^ These principles are incorporated into contemporary ESGO/ESTRO/ESP and ESMO adjuvant treatment algorithms.^(19,25,26,30)^

Despite its clinical relevance, the implementation of molecular classification remains limited by variability in access to testing, cost, and logistical constraints. Ongoing research aims to refine the prognostic and predictive value of molecular subgroups, optimize integration with surgical decision-making, and expand the identification of actionable biomarkers. As molecular classification becomes increasingly standardized and accessible, it is expected to play a central role in precision oncology for endometrial cancer, reducing both overtreatment and undertreatment and improving patient outcomes.^(1,24,33)^

## When should genetic evaluation be indicated in patients with endometrial cancer?

Genetic evaluation is indicated in all patients with suspected or confirmed endometrial cancer through universal screening for Lynch syndrome, using tumor-based mismatch repair (MMR) immunohistochemistry with reflex MLH1 promoter methylation testing when loss of MLH1 is identified, followed by germline testing as appropriate.^(1,6,17,34,35)^ This strategy is recommended regardless of age, histologic subtype, or family history, as approximately 3% of endometrial cancers are associated with Lynch syndrome, and identification has significant implications for patient management and cascade testing of at-risk relatives.^(1,6,35,36)^

Comprehensive genetic counseling and germline testing should be strongly considered in patients diagnosed at a young age (particularly <50 years), those with a personal or family history suggestive of Lynch-associated malignancies (including colorectal, ovarian, gastric, or urothelial cancers), or in the presence of clinical features consistent with other hereditary cancer syndromes, such as Cowden syndrome.^(6,37,38)^ In selected cases, multigene panel testing may be appropriate; however, clinical testing should prioritize genes with established associations with endometrial cancer, particularly mismatch repair genes and PTEN.^(39,40)^

Identification of a pathogenic germline variant should prompt referral for genetic counseling and implementation of cascade testing for family members, enabling individualized surveillance and risk-reducing strategies.^(1,6,35,36)^

Molecular tumor classification, including assessment of POLE mutations, p53 abnormalities, and hormone receptor status, is recommended for prognostic stratification and therapeutic decision-making. However, its role in guiding hereditary cancer risk assessment beyond Lynch syndrome remains limited and continues to evolve.^(17,39,40)^

## What is the preferred surgical approach in apparent early-stage endometrial cancer?

Minimally invasive TH/BSO is the preferred surgical approach for patients with apparent early-stage endometrial cancer. Laparoscopic or robotic-assisted techniques are recommended as the standard of care, as they provide oncologic outcomes equivalent to open surgery while significantly reducing perioperative morbidity, blood loss, length of hospital stay, and postoperative complications. This recommendation is consistently supported by randomized controlled trials, meta-analyses, and large population-based studies, and is endorsed by major international guidelines.^(1,41–43)^

The oncologic safety of minimally invasive surgery in early-stage endometrial cancer has been well established, with no significant differences in overall survival, disease-free survival, or recurrence rates when compared with laparotomy. These benefits are observed across different minimally invasive platforms, including conventional laparoscopy and robotic-assisted surgery, and extend to patients with obesity and relevant comorbidities, in whom minimally invasive approaches significantly reduce surgical morbidity.^(41–44)^

The standard surgical procedure consists of total hysterectomy with bilateral salpingo-oophorectomy. According to the NCCN Guidelines, ovarian preservation may be considered in carefully selected young, premenopausal patients with low-grade, early-stage endometrioid carcinoma and no evidence of extrauterine disease, following individualized counseling and shared decision-making.^(5)^ Vaginal hysterectomy may be an alternative for patients with significant comorbidities or limited surgical fitness who are not candidates for abdominal or minimally invasive approaches, provided that adequate staging can be ensured.^(1,45)^

Robotic-assisted surgery represents an important evolution within minimally invasive techniques for endometrial cancer. Compared with conventional laparoscopy, robotic platforms offer enhanced three-dimensional visualization, improved ergonomics, tremor filtration, and increased instrument articulation, which may facilitate complex pelvic dissections, particularly in patients with obesity, narrow pelvis, or significant anatomic or clinical complexity.^(7,46–48)^

Evidence from systematic reviews, network meta-analyses, and large cohort studies demonstrates that robotic-assisted hysterectomy is associated with reduced blood loss, lower conversion rates to laparotomy, fewer intraoperative and postoperative complications, and shorter hospital stay compared with open surgery, while maintaining oncologic outcomes equivalent to both laparoscopic and open approaches, including overall and disease-free survival.^(7,46–50)^ These advantages are particularly pronounced in obese and high-risk patients, in whom robotic-assisted surgery may expand access to minimally invasive treatment without compromising oncologic safety.^(47–50)^

Based on the available evidence and international consensus, minimally invasive surgery, either laparoscopic or robotic-assisted, should be offered to all eligible patients with apparent early-stage endometrial cancer and should be considered the standard surgical approach when performed by appropriately trained surgeons in experienced centers.^(1,41–43)^

## What is the current role of lymph node assessment in endometrial cancer?

Lymph node assessment plays a central role in the surgical management of endometrial cancer by providing accurate staging, prognostic information, and guidance for adjuvant treatment decisions, rather than offering a direct therapeutic benefit. The indication for nodal assessment is strongest in patients with intermediate- and high-risk clinicopathologic or molecular features, deep myometrial invasion, or non-endometrioid histology, in whom the risk of lymph node metastasis is clinically relevant.^(1,6,9)^

Sentinel lymph node (SLN) mapping is currently the preferred method for lymph node assessment in patients with apparent early-stage endometrial cancer, including those with high-risk histologic subtypes. SLN mapping, most commonly performed using cervical injection of indocyanine green, demonstrates high detection rates and excellent sensitivity and negative predictive value, while significantly reducing surgical morbidity compared with systematic pelvic and para-aortic lymphadenectomy.^(1,6,51–54)^ Bilateral mapping is recommended, and side-specific lymphadenectomy should be performed when mapping fails in a hemipelvis, in accordance with standardized SLN algorithms.^(5,51,54)^

Systematic pelvic and para-aortic lymphadenectomy is no longer recommended for patients with apparent uterine-confined disease, as randomized trials and meta-analyses have consistently shown no improvement in overall or disease-free survival, accompanied by a substantial increase in perioperative morbidity, including lymphedema and lymphocyst formation.^(6,8,9)^ Lymphadenectomy may still be considered in selected cases when SLN mapping is unsuccessful or when grossly suspicious lymph nodes are identified intraoperatively.^(51,54)^

The results of lymph node assessment directly influence FIGO staging and adjuvant treatment recommendations, particularly the indication for chemotherapy and/or radiotherapy in node-positive disease. Pathologic ultrastaging of sentinel lymph nodes improves the detection of low-volume nodal metastases and contributes to more accurate staging, especially in patients with high-risk features.^(51,53,55)^

Sentinel lymph node mapping represents the standard approach to lymph node assessment in endometrial cancer, providing high diagnostic accuracy with significantly lower morbidity than systematic lymphadenectomy, and should be incorporated into surgical staging whenever feasible.^(1,5,6,9,51,53)^

## When can fertility-sparing treatment be considered in patients with endometrial cancer?

Fertility-sparing treatment may be considered in highly selected patients with endometrial cancer who are young, premenopausal, have a strong desire to preserve fertility, and present with strictly low-risk disease. Eligible patients should have grade 1 endometrioid histology, stage IA disease confined to the endometrium, with no evidence of myometrial invasion, lymphovascular space invasion, or extrauterine disease, including synchronous ovarian or adnexal involvement.^(1,56,57)^ Careful pre-treatment assessment with pelvic magnetic resonance imaging or expert transvaginal ultrasonography is mandatory to exclude myometrial invasion and extrauterine spread.^(1,58)^

Fertility-sparing management is contraindicated in patients with high-grade tumors, non-endometrioid histologies (including serous and clear cell carcinoma), or molecular features associated with aggressive behavior, such as p53 abnormality. Candidate selection should involve comprehensive counseling regarding oncologic risks, the need for strict surveillance with serial endometrial sampling, and the recommendation for definitive hysterectomy once childbearing is completed.^(1,57,59–61)^ Although emerging data suggest that molecular and biomolecular markers may further refine patient selection, their routine use to guide fertility-sparing decisions is not yet standardized in clinical practice.^(61,62)^

The preferred fertility-sparing treatments include high-dose oral progestins (medroxyprogesterone acetate or megestrol acetate), a levonorgestrel-releasing intrauterine device, or a combination of both, with or without hysteroscopic tumor resection. These approaches achieve the highest complete response rates in strictly selected patients; however, recurrence remains common, and patients should be counseled accordingly. Pregnancy rates after conservative management are lower than in the general population, and assisted reproductive technologies are frequently required to optimize reproductive outcomes.^(1,57,59,63–65)^

Fertility-sparing treatment is not considered standard of care for patients with grade 2 or higher tumors, myometrial invasion, or non-endometrioid histology. In exceptional cases, conservative management may be discussed within a multidisciplinary setting after individualized risk assessment, but patients must be informed that long-term oncologic safety data remain limited.^(61,65)^

Fertility-sparing treatment in endometrial cancer should be restricted to carefully selected patients with strictly low-risk disease, managed in specialized centers with multidisciplinary expertise, and followed by close surveillance and definitive surgical treatment after completion of childbearing.^(1,5,56–59)^

## How should patients who are not candidates for surgery be managed?

Patients with endometrial cancer who are not candidates for surgery due to advanced age, significant comorbidities, poor performance status, or patient refusal should be managed through a multidisciplinary, individualized approach. Treatment selection should be guided by disease stage, histology, molecular profile, and patient-related factors, with the primary goals of disease control, symptom relief, and preservation of quality of life.^(1,2,5,66)^

Radiation therapy represents the main curative or definitive treatment option for non-surgical candidates with early-stage or locally advanced disease. External beam radiotherapy, vaginal brachytherapy, or a combination of both can achieve effective local control, with brachytherapy associated with lower toxicity in appropriately selected patients. In carefully selected early-stage cases, oncologic outcomes may approach those of surgery; however, cure rates are generally lower, particularly in more advanced disease.^(1,2,66)^ In unresectable or locally advanced tumors, radiotherapy may be combined with systemic therapy to improve disease control.^(1,6)^

Hormonal therapy is an appropriate option for patients with low-grade, estrogen and/or progesterone receptor–positive tumors, particularly in frail or elderly patients or those with limited life expectancy. Commonly used agents include oral progestins (medroxyprogesterone acetate or megestrol acetate), levonorgestrel-releasing intrauterine systems, and, in selected cases, aromatase inhibitors or tamoxifen. Response rates are highest in well-differentiated, hormone receptor-positive tumors, although responses are often incomplete and recurrence is common.^(1,6,59,67,68)^

For patients with advanced or recurrent disease who are not surgical candidates, systemic therapy is the standard of care. Platinum-based chemotherapy, typically carboplatin plus paclitaxel, remains the backbone regimen. The incorporation of immune checkpoint inhibitors into first-line therapy has significantly improved progression-free and overall survival in advanced or recurrent endometrial cancer.^(1,6,69)^ Molecular profiling is essential to guide treatment selection: immune checkpoint inhibitors are indicated for mismatch repair–deficient or microsatellite instability–high tumors, while the combination of lenvatinib and pembrolizumab is recommended for mismatch repair–proficient tumors after progression on prior therapy. In patients with HER2-positive uterine serous carcinoma, trastuzumab should be added to platinum-based chemotherapy.^(1,6)^

Supportive and palliative care should be integrated early in the management of non-surgical candidates, particularly in advanced disease. Symptom control, psychosocial support, and optimization of quality of life are essential components of care and should be addressed alongside oncologic treatment.^(2,70)^

In summary, management of patients with endometrial cancer who are not candidates for surgery should be individualized and based on a combination of radiotherapy, systemic therapy, hormonal treatment, and supportive care, guided by tumor biology, disease extent, and patient preferences within a multidisciplinary framework.^(1,2,6,66)^

## What is the role of surgery in advanced or recurrent endometrial cancer?

Surgery in advanced or recurrent endometrial cancer should be considered only in carefully selected patients and within a multidisciplinary framework. Surgical candidacy depends on performance status, comorbidities, disease distribution, prior treatments, and, most importantly, the likelihood of achieving complete cytoreduction with acceptable morbidity.^(1,71,72)^

In patients with advanced-stage disease (FIGO stage III–IV), primary cytoreductive surgery may be considered when complete resection to no gross residual disease is deemed feasible. Achieving complete cytoreduction is consistently associated with improved survival, whereas suboptimal debulking offers limited benefit. When complete resection is unlikely or surgical risk is excessive, neoadjuvant chemotherapy followed by interval cytoreductive surgery represents an alternative strategy, with lower perioperative morbidity and outcomes comparable to upfront surgery with residual disease.^(1,71,72)^

For patients with recurrent endometrial cancer, surgery may be appropriate in selected cases with isolated or limited disease, long disease-free interval, and good performance status. Secondary cytoreductive surgery, including non-exenterative procedures, is associated with prolonged post-recurrence survival when optimal resection is achieved. Pelvic exenteration may be considered in highly selected patients with central pelvic recurrence and no evidence of extrapelvic disease, with curative intent in rare cases.^(1,70,73,74)^ Surgical decision-making should account for recurrence site, extent of disease, prior radiotherapy, and overall disease biology.

Palliative surgical interventions may be indicated for symptom control in advanced or recurrent disease, including management of bowel obstruction, fistulas, or uncontrolled bleeding, with the primary goal of improving quality of life rather than prolonging survival.^(1,72)^

Surgery in advanced or recurrent endometrial cancer should be integrated with systemic and locoregional therapies. Chemotherapy, radiotherapy, and targeted or immune-based treatments constitute the backbone of management in these settings, and surgical interventions should complement, not delay, effective systemic treatment. Molecular profiling, including assessment of mismatch repair status, p53 abnormalities, hormone receptor expression, and HER2 status in serous tumors, informs systemic therapy selection but currently plays a limited role in determining surgical candidacy.^(6,17,71)^

In summary, surgery in advanced or recurrent endometrial cancer is not routinely indicated and should be reserved for carefully selected patients in whom complete cytoreduction or meaningful symptom palliation is achievable. Optimal outcomes depend on meticulous patient selection, multidisciplinary decision-making, and integration with modern systemic therapies.^(1,71,72,74)^

Key practical recommendations and common pitfalls in the surgical management of endometrial cancer are summarized as do's and don'ts in figure 4.

**Figure 4:**
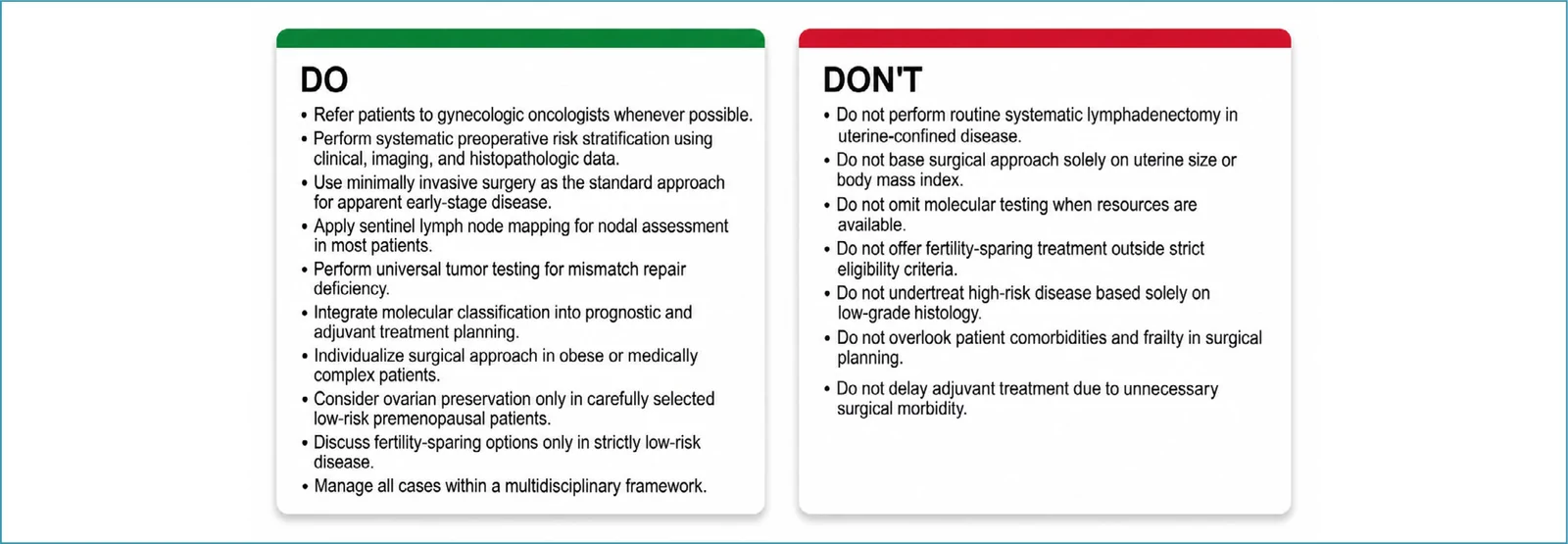
Do's & Don'ts in the surgical management of endometrial cancer

## Final considerations

Surgery remains the cornerstone of endometrial cancer management and plays a central role in staging, prognostic stratification, and treatment planning. Advances in minimally invasive surgery, sentinel lymph node mapping, and molecular classification have shifted surgical practice toward more precise, risk-adapted approaches that preserve oncologic safety while reducing morbidity. Minimally invasive hysterectomy with bilateral salpingo-oophorectomy is now the standard of care for apparent early-stage disease, and sentinel lymph node mapping has replaced routine lymphadenectomy for most patients. Surgical decision-making should be individualized, integrating clinicopathologic and molecular factors, patient comorbidities, and reproductive goals, and supported by multidisciplinary care. This Position Statement summarizes current evidence and provides practical guidance on the surgical management of endometrial cancer, aiming to promote high-quality, consistent, and patient-centered surgical practice while allowing flexibility in the context of evolving scientific knowledge.

## Data Availability

research data are available in the article

## National Specialized Commission on Gynecologic Oncology of the Brazilian Federation of Gynecology and Obstetrics Associations (Febrasgo)

President:

Eduardo Batista Cândido

Vice-president:

Renato Moretti Marques

Secretary:

Sophie Françoise Mauricette Derchain

Members:

Ana Carla Franco Ubinha
Caetano da Silva Cardial
Cristina Anton
Delzio Salgado Bicalho
Etelvino de Souza Trindade
Geórgia Fontes Cintra
Jesus Paula Carvalho
Jose Carlos de Jesus Conceição
Leandro Santos de Araújo Resende
Marcia Luiza Appel Binda
Mariana Seabra Leite Praça
Ricardo dos Reis
Suzana Arenhart Pessini
Walquiria Quida Salles Pereira Prim
